# Case report: Ultrasound-guided percutaneous drainage combined with lavage using urokinase: An economical and effective treatment for muscular hematomas in hemophiliacs

**DOI:** 10.3389/fsurg.2023.1023329

**Published:** 2023-03-24

**Authors:** Hao Liu, Cong Xu, Weizhen Wang, Bin Chen, Jing Sun, Xiaoqin Feng, Yaru Zhang, Fei Ma, Lingli Du, Yang Gao, Yingjia Li

**Affiliations:** ^1^ Department of Ultrasound, Nanfang Hospital, Southern Medical University, Guangzhou, China; ^2^ Deportment of Orthopaedics and Traumatology, Nanfang Hospital, Southern Medical University, Guangzhou, China; ^3^ Department of Hematology, Nanfang Hospital, Southern Medical University, Guangzhou, China; ^4^ Department of Pediatrics, Nanfang Hospital, Southern Medical University, Guangzhou, China

**Keywords:** hemophilia, hematoma, interventional ultrasonography, urokinase, percutaneous drainage

## Abstract

This was an initial effort to treat hemophiliac hematoma by ultrasound-guided intratumoral drainage and lavage with urokinase after adequate supplementation of coagulation factors. Two patients with severe hemophilia underwent ultrasound-guided percutaneous drainage in combination with lavage using urokinase. After 5-day and 3-day treatments, respectively, intramuscular hematomas in both patients disappeared, compression symptom was relieved, and no obvious adverse reactions or serious complications were observed during the treatment or follow-up. These findings suggest that ultrasound-guided drainage combined with lavage using urokinase is an immediate, safe, effective, and minimally invasive treatment for intramuscular hematomas in hemophiliacs, avoiding potential complications by surgical resection with relatively low treatment cost.

## 1. Introduction

Hemophilia is a hereditary disorder due to the deficiency of coagulation factors. Patients with severe hemophilia may develop spontaneous or post-traumatic bleeding, with 10%–25% of the bleeding occurring in muscles. If the bleeding is not treated promptly, muscular hematoma could lead to serious complications, including nerve injury, osteofascial compartment syndrome, and sepsis. The World Federation of Hemophilia recommends treatment with high-dose coagulation factors for managing muscular hematomas in hemophiliacs (1). However, in China and other developing countries, patients with hemophilia often have limited access to coagulation factor replacement therapy due to economic constraints, resulting in a higher incidence of muscular hematoma and a guarded prognosis.

Ultrasound-guided percutaneous drainage is a fast, real-time, and well-tolerated procedure that could remove hematomas in a short time. However, in hemophilia cases, repeated bleeding may result in organized viscous hematomas, potentially lowering the success of drainage of the contents. It was reported that urokinase, a thrombolytic enzyme, could degrade fibrin, disperse hematoceles, and ease drainage in hematomas (2). Hence, we describe our initial effort to treat muscular hematomas in hemophiliacs by ultrasound-guided percutaneous drainage and lavage using urokinase after enough coagulation factor therapy.

## 2. Case description

**Figure 1:**
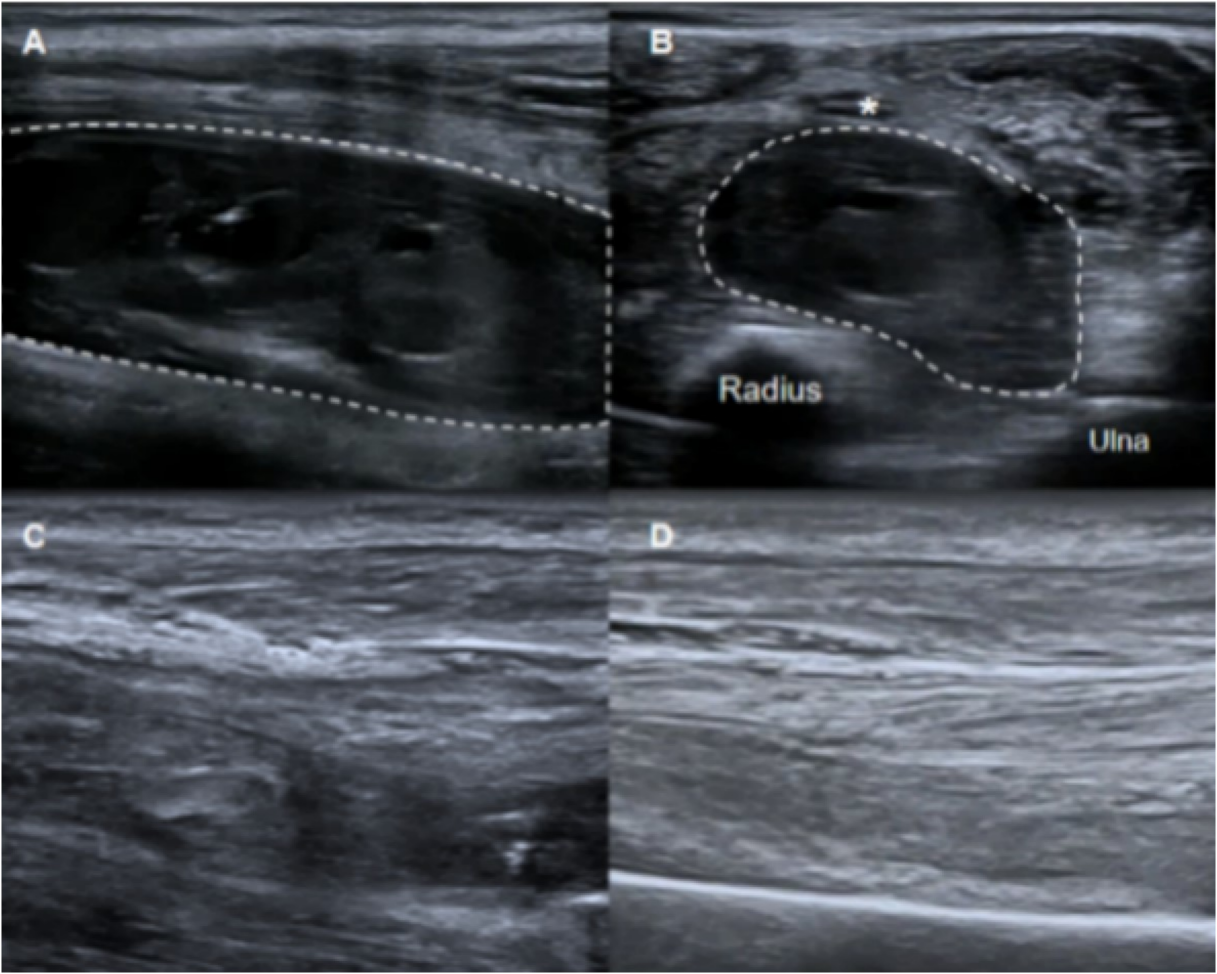
A bone traction device was fastened to the patient's right leg (**A**). Ultrasound examination revealed a heterogeneous echo mass in the lateral muscles of the right calf (**B**). A 12-F drainage tube was inserted into the hematoma (**C**). Five days after drainage and urokinase lavage, ultrasonography showed significantly decreased hematoma volume (**D**).

Patient A, a middle-aged man with severe hemophilia B, underwent orthopedic surgery because of severe bone destruction and decreased muscle function of the right knee caused due to repeated bleeding. The patient had progressive swelling on the right leg, accompanied by reduced sensation from the right leg to the foot dorsum three weeks later. Ultrasound examination revealed a heterogeneous mass on the lateral muscles of the right calf with an approximate size of 17 × 8 × 7 cm^3^. It was concluded from clinical symptoms and ultrasonographic features that the movement disorder and sensory disturbance in the right leg resulted from compression by the intramuscular mass. To examine the composition of the mass, a 2.4 ml bolus of contrast agent (SonoVue; Bracco Diagnostic Inc., Milano, Italy) was injected into the elbow vein before ultrasonography. Circular hyperenhancement was observed at the periphery while no enhancement was seen at the center, suggesting that the mass was a hematoma with no granulation or neovascularization (Supplementary Figure S1). Following verification, the muscular hematoma was drained under ultrasound guidance. After administration of recombinant human coagulation factor VIII at a dose of 1,500 IU, a puncture needle (16G) was successfully inserted into the hematoma under ultrasound guidance, but no hematocele could be drained. Then, a 0.038-inch wire was placed into the hematoma through the needle, followed by the insertion of a 12-F drainage tube. However, no hematocele was still drained due to the highly viscous nature of the fluid. Lavage using urokinase was then performed since there was no obvious granulation tissue or neovascularization within the hematoma based on the contrast-enhanced ultrasound (CEUS) images. To drain the viscous hematocele, 30,000 IU of urokinase was dissolved in physiological saline (20 ml) and injected into the hematoma through the drainage tube. Subsequently, the drainage tube was clamped for 6 h to allow for thrombolysis and then drained for 2 h. These procedures were repeated twice daily. The procedure was carefully monitored and the urokinase lavage was modified if the drainage fluid turned pale pink. To maintain factor VIII activity above 30% during this procedure, the patient was prescribed prothrombin complex twice daily at 25 IU/kg. The changes in muscular hematoma were monitored by ultrasound. The hematoma gradually shrank (volume was estimated based on length × width × height × 0.52 by ultrasound) during the five-day treatment. Three months later, no recurrence of the muscular hematoma was observed on ultrasonographic images, and the function of the lower limbs gradually recovered (Figure 1).

**Figure 2:**
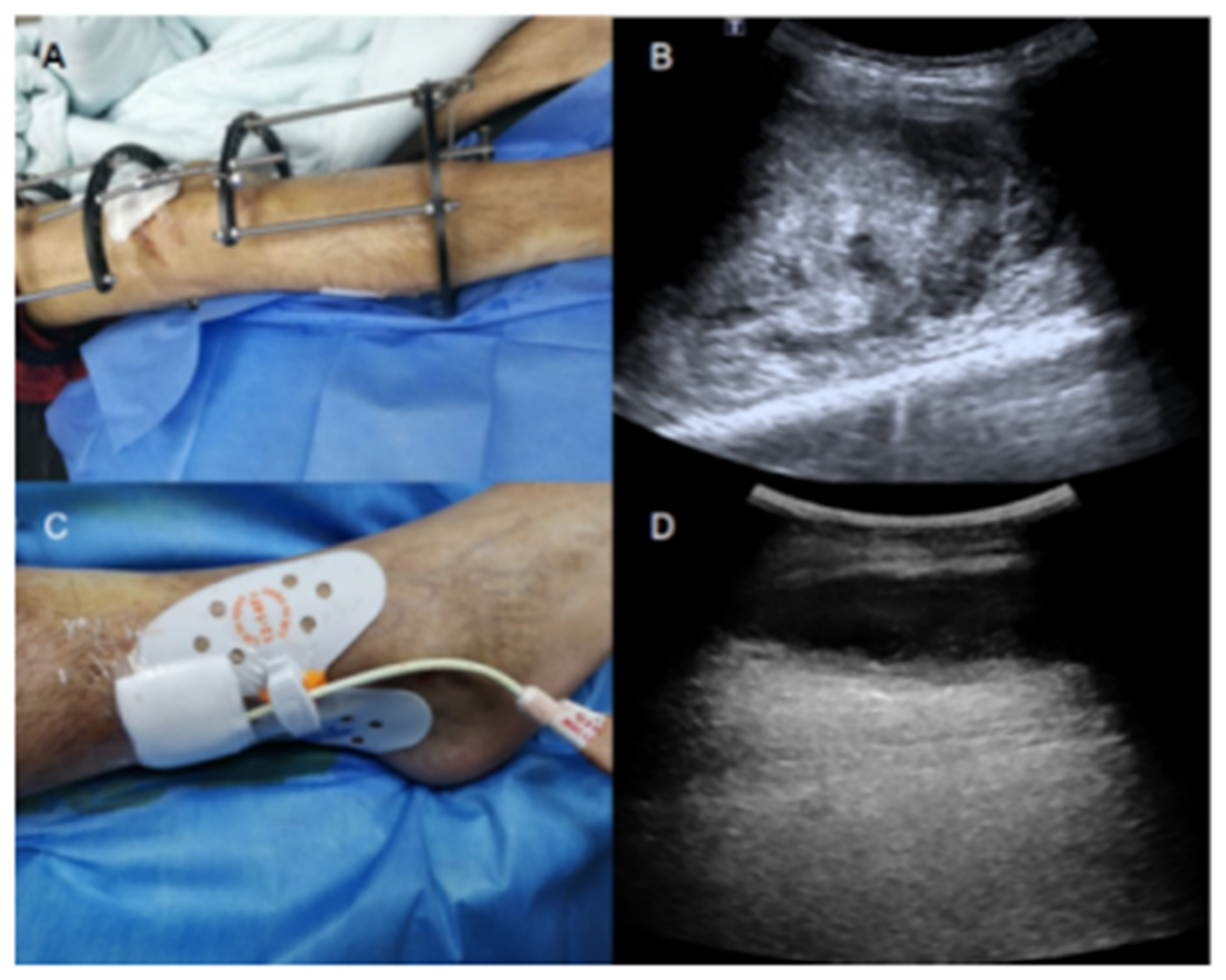
Ultrasound examination revealed a polycystic hematoma in the forearm flexors (long axis section of the hematoma; (**A**). The hematoma was located between the median nerve (*), and radius-ulna (short axis section of the hematoma; (**B**). Three days after drainage and urokinase lavage, ultrasonography showed that the hematoma nearly disappeared (**C**). Hematoma did not recur at the 4-month follow-up after treatment (**D**).

Patient B, a 27-year-old man with severe hemophilia A, observed gradual swelling of his right forearm two weeks ago. One week later, the patient felt numbness in the right thumb, index finger, and middle finger, occasionally accompanied by mobility disorder. Ultrasonographic examination revealed a polycystic hematoma in the flexors of the forearm. The hematoma was located between the ulna-radius and median nerve with an approximate size of 10.5 × 3.5 × 3.2 cm^3^, compressing the median nerve, which was swollen and thickened on the ultrasound images. CEUS was performed after injecting a 2.4 ml bolus of contrast agent (SonoVue, Bracco Diagnostic Inc.) into the elbow vein, but the no-enhanced area was found inside the hematoma (Supplementary Figure S2). The patient had normal coagulation function and produced no factor VIII inhibitors. The ultrasound-guided percutaneous catheter drainage was performed to relieve the compression on the median nerve. A 14-F drainage tube with several side holes was placed along the long axis of the hematoma to penetrate the three main capsules with ultrasound guidance. After administration of coagulation factor (20 IU/kg, twice daily), a 20 ml saline solution containing 30,000 IU of urokinase was injected *via* the drainage tube, which was clamped for 6 h and then drained for 2 h. This procedure was repeated twice daily. The hematoma disappeared as confirmed by ultrasound examination three days after the procedure. The patient gradually recovered from movement disorder and sensation disturbance in the right forearm. No recurrence of muscular hematoma was observed after four months (Figure 2).

This study was approved by the ethics committee of our hospital. Written informed consent was obtained from both patients before conventional ultrasound and CEUS examinations.

## 3. Discussion

The most widely accepted treatment option for muscular hematoma in hemophiliacs is coagulation factor replacement therapy (1). However, many patients with hemophilia in China cannot afford the long-term use and/or high dose of coagulation factors. Additionally, this treatment does not immediately reduce the size of the hematoma. Surgical resection of the hematoma brings a high risk of bleeding in patients with hemophilia, limiting its application. Ultrasound-guided percutaneous drainage is an immediate, low-cost, minimally invasive method for the treatment of muscular hematoma in hemophiliacs, but its effectiveness is questionable when muscular hematomas resulting from repeated bleeding are too viscous for effective draining (2–4). Urokinase that degrades fibrin in blood clots dissolves the viscous contents of hematomas by intracavitary lavage such as intracranial, subdural, and retroperitoneal lavages (5–7). Therefore, we firstly attempted to perform ultrasound-guided drainage combined with lavage using urokinase to treat large muscular hematomas in hemophilia cases.

In patient A, a large intramuscular hematoma was present and ultrasound-guided percutaneous drainage was performed to remove the hematoma. The large hematomas are usually removed by surgery. The operation principle is extensive fasciotomy, which may lead to massive trauma, hemorrhage, scar contracture, and other sequelae. Peripheral neuropathy and loss of function may easily occur if the hematoma is not treated promptly. Therefore, this drainage and lavage procedure is more suitable when there is a large hematoma. Additionally, only about 400 IU/kg coagulation factor was administered during the treatment. According to previous reports, for muscular hematomas of similar sizes, the coagulation factor dose used for the replacement therapy could be as high as 3,000–4,000 IU/kg, and the treatment could last for more than 20 days. In comparison with coagulation factor replacement therapy and surgery, ultrasound-guided drainage combined with lavage using urokinase is a feasible option in hemophiliacs in economically underdeveloped areas due to excellent performance in evacuating large muscular hematomas in a minimally invasive manner and reducing coagulation factor dosage and treatment costs.

In patient B, although the muscular hematoma was relatively small, its removal was urgent in order to relieve the compression of the median nerve. The patient underwent ultrasound-guided drainage combined with lavage using urokinase, and the hematoma shrank significantly after a three-day treatment. The factor VIII activity was maintained above 30% during this procedure.

Urokinase can be used to dissolve acute (1–14 days) and subacute (15–28 days) thromboses or hematomas effectively (8). Fortunately, the hematoma formation times in these two cases were within four weeks. Urokinase application was a good treatment option for dissolving the hematoma in both cases because no obvious granulation tissue or neovascularization were formed inside the hematoma as assessed by CEUS, which minimized the risk of hemorrhage caused by urokinase entering the circulation. Moreover, the urokinase dosage should not be too large to avoid or reduce bleeding complications (9). Although only two cases are presented in this manuscript, both of them were carefully monitored during the low-dose urokinase application, and the urokinase lavage was modified if the volume and color of the drainage fluid was changed.

With this treatment, the hematoma was drained effectively, and rebleeding didn't occur during the subsequent 3-month follow-up. In the future, more trials are needed to determine the appropriate urokinase dosages for different volumes of hematomas to ensure the treatment effectiveness.

## 4. Conclusion

Ultrasound-guided drainage combined with lavage using urokinase is a minimally invasive therapeutic option for intramuscular hematomas in hemophiliacs. The procedure relieved the pressure from the hematoma immediately, shortened the treatment period, reduced the consumption of the coagulation factor, and limited the total cost. However, further optimization is required to determine the appropriate dosages and effective times of urokinase lavage application for hematomas of different sizes at various locations. In addition, the sample size was two cases and the follow-up duration was relatively short. Further large-scale clinical studies with long-term follow-up are warranted to confirm the safety and efficacy of this method for the treatment of muscular hematoma in hemophiliacs.

## Data Availability

The original contributions presented in the study are included in the article/**Supplementary Material**, further inquiries can be directed to the corresponding author.
