# Supplementary material for: Case report: Ultrasound-guided percutaneous drainage combined with lavage using urokinase: An economical and effective treatment for muscular hematomas in hemophiliacs
Source: Front Surg. 2023 Mar 24;10:1023329. doi: 10.3389/fsurg.2023.1023329 (PMC10079870; doi:10.3389/fsurg.2023.1023329)
Supplement: Supplementary file 1 [file Datasheet1.docx]

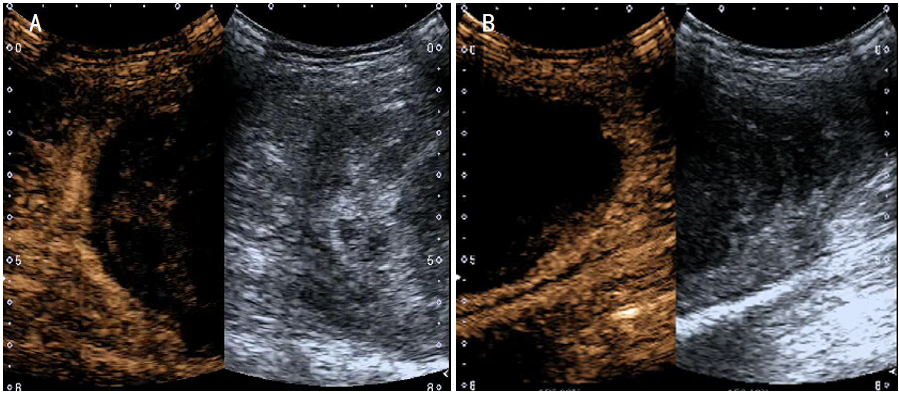


Fig. S1. Contrast-enhanced ultrasonography of hematoma (A. cephalic side of hematoma; B. foot side of hematoma) Circular hyperenhancement was observed in the periphery while no enhancement was found in the central space, suggesting that no obvious granulation tissue and neovascularization were formed inside the hematoma.


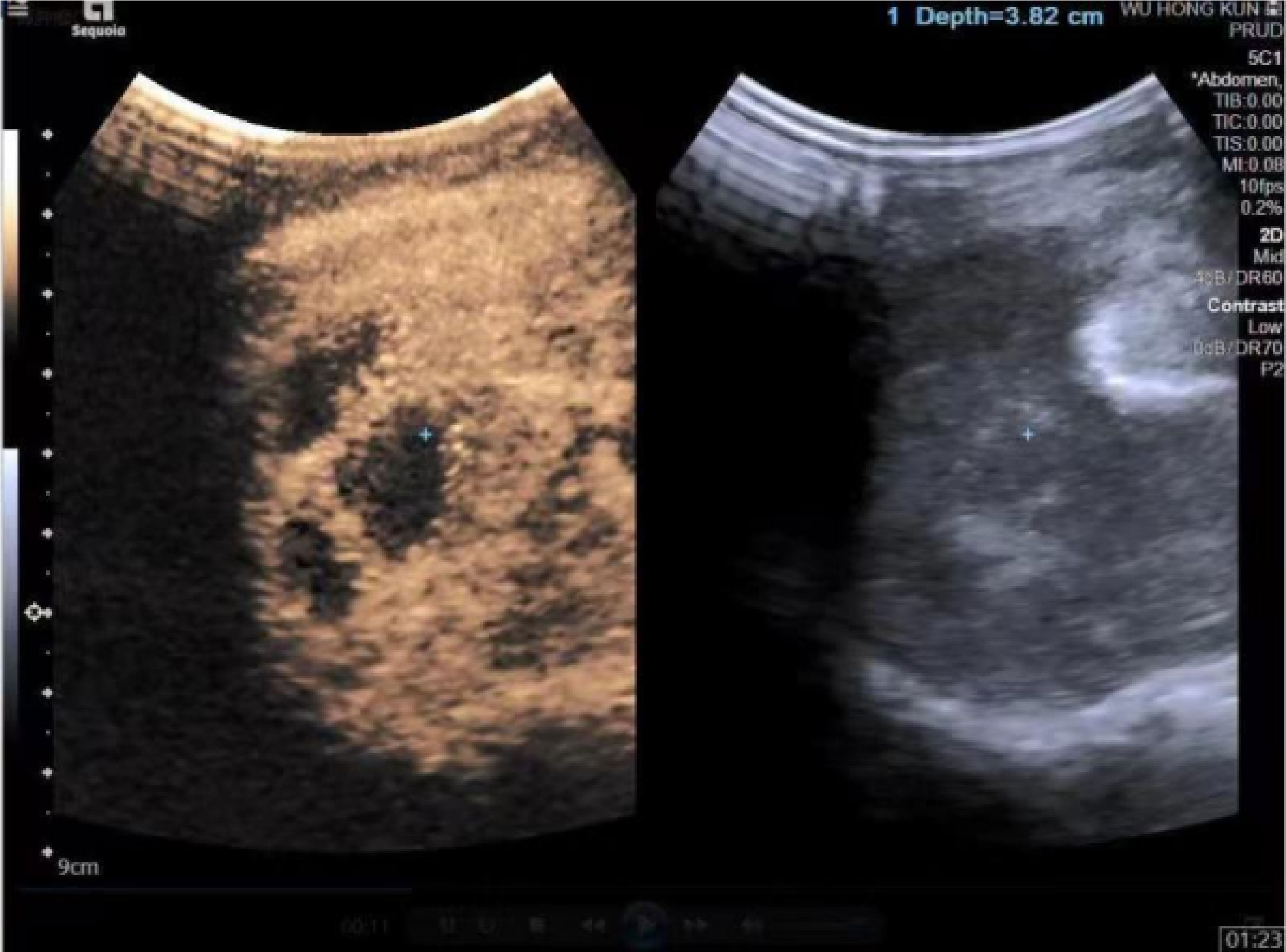


Figure S2. Contrast-enhanced ultrasonography of hematoma. No-enhanced area was found inside the hematoma, suggesting that no obvious granulation tissue and neovascularization were formed inside the hematoma.


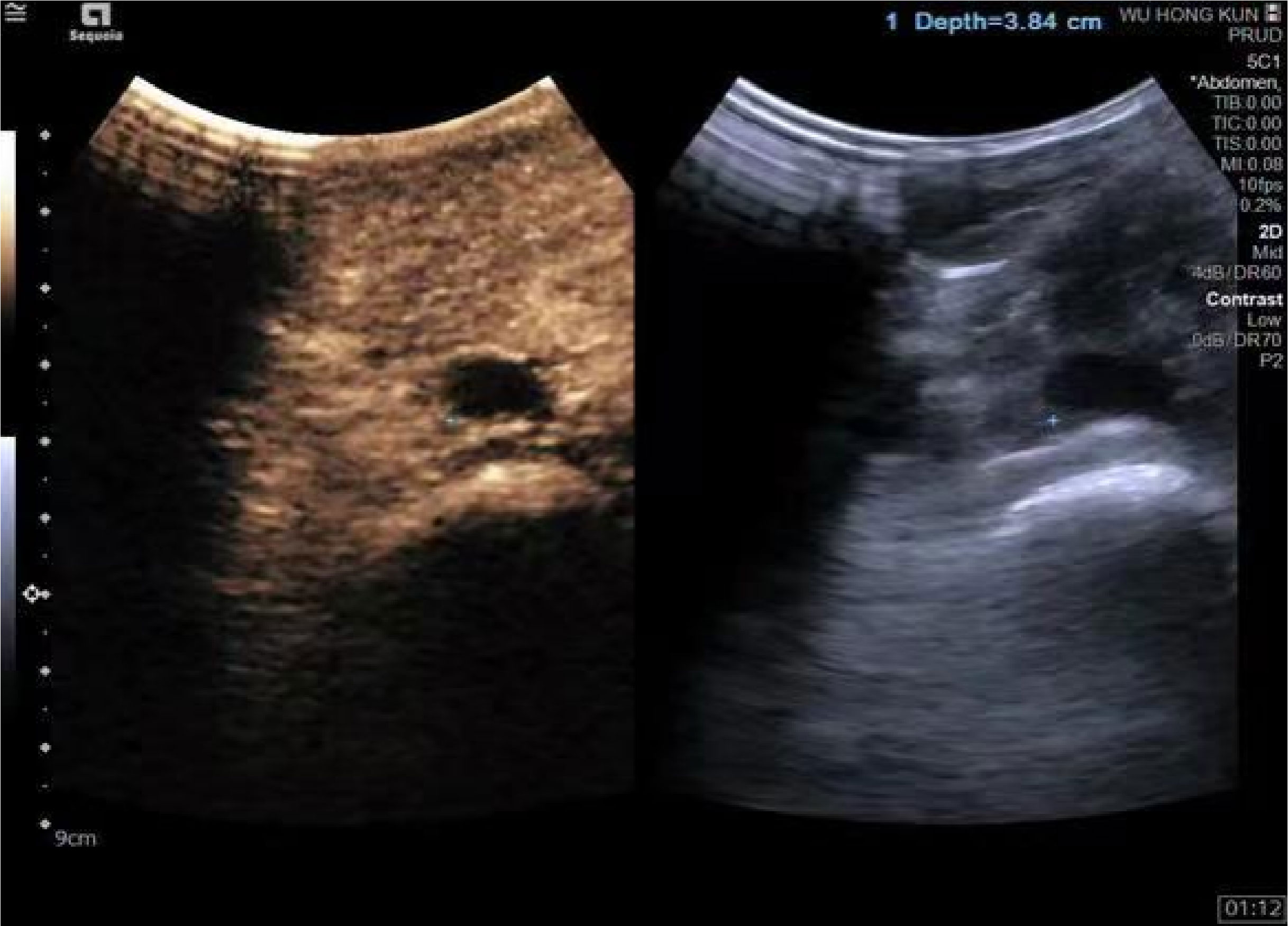


Figure S3. Contrast-enhanced ultrasonography revealed an uneven hyperenhancement in the mass with scattered no-enhanced areas, suggesting that the mass might be a hematoma with abundant granulation tissue and neovascularization formed inside.
