# Supplementary material for: Case report: Ultrasound-guided percutaneous drainage combined with lavage using urokinase: An economical and effective treatment for muscular hematomas in hemophiliacs
Source: Front Surg. 2023 Mar 24;10:1023329. doi: 10.3389/fsurg.2023.1023329 (PMC10079870; doi:10.3389/fsurg.2023.1023329)
Supplement: Supplementary file 2 [file Datasheet2.docx]

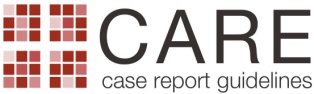
CARE Checklist of information to include when writing a case report
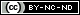


**Topic Item Checklist item description Reported on Line**

**Title 1** The diagnosis or intervention of primary focus followed by the words “case report” P1 :Title line2-3

**Key Words 2** 2 to 5 key words that identify diagnoses or interventions in this case report, including "case report" P1 :L16-17

# Abstract

**(no references)**

**3a** Introduction: What is unique about this case and what does it add to the scientific literature? P1 :L19-21

**3b** Main symptoms and/or important clinical findings P1 :L21-23

**3c** The main diagnoses, therapeutic interventions, and outcomes P1: L23,P2 :L26

**3d** Conclusion—What is the main “take-away” lesson(s) from this case? P2 :L26-29

**Introduction 4** One or two paragraphs summarizing why this case is unique (**may include references**) P2: L31-48

**Patient Information 5a** De-identified patient specific information P2-P4: L50-L52, L82-L83,L102

**5b** Primary concerns and symptoms of the patient P2-P4: L52-L53, L83-L8, :L102

**5c** Medical, family, and psycho-social history including relevant genetic information P2-P4:L54-L55, L85-L88, L103

**5d** Relevant past interventions with outcomes P2:L50-L52

# Clinical Findings

**Timeline**

**Diagnostic Assessment**

**Therapeutic Intervention**

**Follow-up and Outcomes**

1. Describe significant physical examination (PE) and important clinical findings P2:L54-L55, P3:L85-L88, P4 L103
2. Historical and current information from this episode of care organized as a timeline P2-P3: L52, L83

**8a** Diagnostic testing (such as PE, laboratory testing, imaging, surveys). P2-P4:L54-L55, L85-L88, L103

**8b** Diagnostic challenges (such as access to testing, financial, or cultural) P2-P4: L58-L60, L88-L90, L104-L105

**8c** Diagnosis (including other diagnoses considered) P2-P4: L61-L62,L87-L88,L106=L107

**8d** Prognosis (such as staging in oncology) where applicable NA

**9a** Types of therapeutic intervention (such as pharmacologic, surgical, preventive, self-care) P2-P4:L63.L91-L92, L108

**9b** Administration of therapeutic intervention (such as dosage, strength, duration) P2-3:L64-76, L93-97

**9c** Changes in therapeutic intervention (with rationale) P2:L66-70

**10a** Clinician and patient-assessed outcomes (if available) NA

**10b** Important follow-up diagnostic and other test results P2-3: L77-80, L98-101

**10c** Intervention adherence and tolerability (How was this assessed?) NA

**10d** Adverse and unanticipated events NA

**Discussion 11a** A scientific discussion of the strengths AND limitations associated with this case report P4-5 :L113-161

**11b** Discussion of the relevant medical literature **with references** P4-5 :L113-161

**11c** The scientific rationale for any conclusions (including assessment of possible causes) P4: L125-126,

**11d** The primary “take-away” lessons of this case report (without references) in a one paragraph conclusion P5:L163-171

**Patient Perspective 12** The patient should share their perspective in one to two paragraphs on the treatment(s) they received NA

**Informed Consent 13** Did the patient give informed consent? Please provide if requested . . . . . . . . . . . . . . . . . . . . . . . . . . . . . . . . . . . . . . **Yes √ No**
